# Industrially Produced Fe- and Mn-Based Perovskites: Effect of Synthesis on Reactivity in Three-Way Catalysis: Part 2

**DOI:** 10.1021/acsomega.1c02132

**Published:** 2021-09-14

**Authors:** Elena Brusamarello, Cataldo Blonda, Cristina Salazar-Castro, Paolo Canu, Antonella Glisenti

**Affiliations:** † Dept. of Chemical Sciences, University of Padova, Via F. Marzolo, 1, 35131 Padova, Italy; ‡ Dept. of Industrial Engineering, University of Padova, Via F. Marzolo, 9, 35131 Padova, Italy; § L’Urederra Foundation, Perguita Industrial Area, No. 1 Street, CP 31210 Los Arcos (Navarra), Spain; ∥ CNR-ICMATE, INSTM, Via F. Marzolo, 1, 35131 Padova, Italy

## Abstract

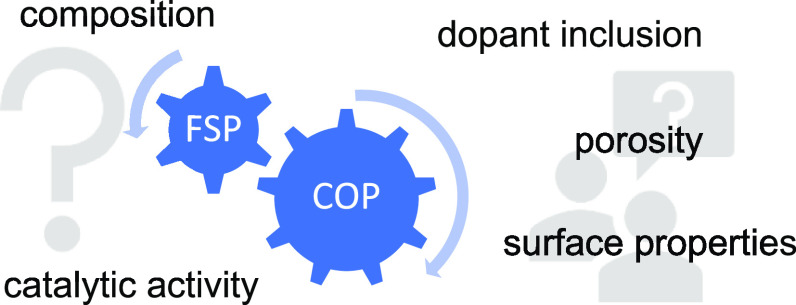

Mn-based perovskites obtained by two different industrial procedures [flame spray pyrolysis (FSP) and co-precipitation (COP)] have been extensively compared in terms of chemical, structural, and morphological properties with the aim of evaluating how the upscale of complex catalysts can affect the functionality. The transition between laboratory and production scale is, in fact, usually not straightforward. The catalytic activity was tested focusing on reactions of relevance in the abatement of pollutants. In particular, CO-assisted NO reduction (which could be also considered as a model reaction) and reactions with a synthetic automotive exhaust mixture, including 10% steam and oxygen, were carried out. The development of three-way catalysts is still a relevant question: noble metal-free, efficient catalysts are even more necessary in hybrid vehicles. For this purpose, the catalytic activity of the samples has been correlated with the characterization results and thus with the peculiar aspects of the production method. Relevant differences have been observed between COP and FSP catalysts, in terms of the specific surface area, surface composition, and presence of surface-active sites. Also, the different efficiencies of inserting dopants in the perovskite unit cell and thus in reducibility and ion mobility are relevant. Despite having the same composition and crystalline structure, the catalytic activity and the effect of pre-treatments are observed to depend on the production procedure.

## 1 Introduction

This paper should be considered as a direct continuation of part 1; the aim is to evaluate if the effect of preparation procedure described and discussed in part 1 is specifically to be intended for ferrites or it can be considered more generally a problem of upscaling of perovskite oxides. The literature data suggest that good activities can be obtained, considering Fe, Co, and Mn in the B site.^1−3^ Cobalt is very active in the abatement of pollutants, with particular reference to oxidation^4−6^ but also activity in reduction can be enhanced through doping and tuning of the nanocomposition.^7,8^ However, its use on the industrial scale is not an easy task; because of this, we focused on Co-doped LaMnO_3_ in which only a minimum amount of cobalt is included.

Mn-based perovskites have already been proven to be promising in the literature^9−13^ in TWC applications. In particular, Mn insertion in perovskite B-site is beneficial, especially for oxidation of hydrocarbons and CO.^14−16^ A-site doping is found to be less decisive; however, partial substitution at the A-site with a cation of different valence (such as K^+^ instead of La^3+^) induces the formation of oxygen vacancies and/or changes in the oxidation state of the B-site cation, increasing the catalytic properties of the materials.^13,15,17^ K^+^ is considered to be able to activate both gas pollutants and soot. Furthermore, work is in course in our laboratories to test also the behavior of K-doped manganites with respect to soot oxidation. For these materials, the commonly accepted mechanism is based on structural defects and vacancies which affect oxygen uptake and release. This phenomenon goes under the name of Mars-van-Krevelen mechanism, and it implies the presence of two distinct types of catalytically active oxygen species in perovskites: suprafacial oxygen species (commonly denoted as α) and intrafacial oxygen (β). At lower temperature (i.e., below 600 °C), suprafacial oxygen is available and reacts, whereas at higher temperature, bulk β oxygen is activated and takes part in the catalytic oxidation, being replenished by dissociation of molecular oxygen (or NO) from the gas phase.^18^

In the first part of the article, we concluded that the preparation approach deeply influences the inclusion extent of the dopants and this leads to some significant consequences: not only activity and selectivity in the catalytic reactions carried out were affected but also morphology, crystallographic structure, and depth compositions were affected. Interestingly, the surface area does not play a relevant factor in determining the activity, but rather, the active oxygen distribution throughout the samples is playing the major role. Co-precipitation (COP) technique is not efficient in allowing Ca inclusion in the lattice and therefore seems less attractive for industrial application, while flame spray pyrolysis (FSP) synthesis allows Ca to enter the structure, and this causes the formation of species like Fe(IV) and oxygen vacancies, an active site for molecule oxidation in the gas phase.

## 2 Experimental Section

### 2.1 Synthesis: FSP and COP

Experimental details can be referred to the ones described in the first part of the article as the same FSP procedures were employed.

For COP, KOH (>85%, 795 g, and 12 mol) was dissolved in water (7 L) and the solution was stirred and heated to 60 °C. Lanthanum nitrate hexahydrate (974.3 g and 2.25 mol), potassium nitrate (25.3 g and 0.25 mol), manganese nitrate (50 wt/w % solution, 805.3 g, and 2.25 mol), and cobalt nitrate hexahydrate (72.8 g and 0.25 mol) were dissolved in water to give 1.5 L total volume of solution. The salt solution was added to the base at 10 mL/min. When the addition was complete, the precipitate was aged with stirring for 30 min at 60 °C. The material was collected by vacuum filtration, washed to remove adsorbed ions, and dried at 105 °C. The sample was calcined at 700 °C for 2 h in air to form the perovskite phase.

### 2.2 Characterization and Activity Tests

Both characterization and catalytic activity tests have been carried out following the same experimental setup and conditions as reported in part 1.

Both extended (survey, 187.85 eV pass energy, 0.5 eV/step, and 0.05 s/step) and detailed (for La3d, O 1s, C 1s, Mn 2p, Co 2p, and K 1s—23.5 eV pass energy, 0.1 eV/step, and 0.1s/step) XP spectra were collected with a standard Al Kα source working at 250 W.

X-ray diffraction (XRD) analyses were performed with a Bruker D8 ADVANCE diffractometer with Bragg–Brentano geometry using Cu Kα radiation (40 kV, 40 mA, and *k* = 0.154 nm).

Field emission-scanning electron microscopy and energy dispersive X-ray spectroscopy measurements were carried out on Zeiss SUPRA 40VP (voltage 20 kV).

Temperature-programmed reduction tests were performed with Micromeritics Autochem II 2920, equipped with a thermal conductivity detector [from room temperature (RT) to 900 °C at 10 °C/min under a constant flow of H_2_ 5% in Ar].

Two series of catalytic activity tests were carried out, at atmospheric pressure: NO + CO mixture (in a stoichiometric ratio and between RT and 500 °C) and with a complex mixture simulating actual conditions of an automotive exhaust (in addition to a larger number of species, 10 vol % of steam and 15 vol % of CO_2_ were always used).

## 3 Results and Discussion

### 3.1 Characterization

The specific surface area of La_0.9_K_0.1_Mn_0.9_Co_0.1_O_3_ prepared by FSP (LKMC FSP) is 64.9 m^2^/g; for the one obtained by COP (LKMC COP) a lower value, 29.0 m^2^/g, has been observed.

**Figure 1:**
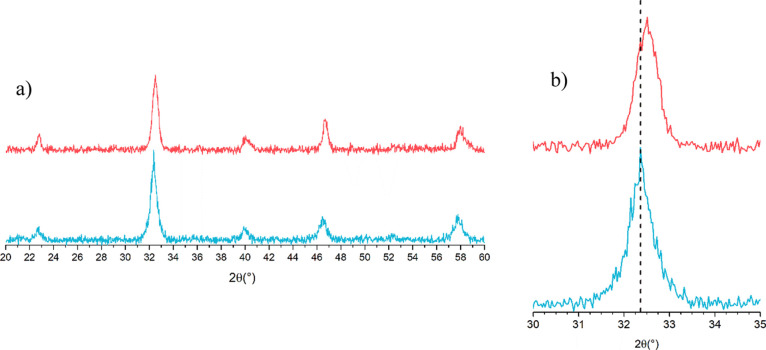
(a) XRD pattern of samples LKMC FSP (blue) and LKMC COP (red). (b) Inset of the most intense peak.

The diffraction patterns (Figure 1) agree with expectations for this perovskite; a slight shift toward lower angles for the diffraction peaks of LKMC COP, suggests a different level of dopant inclusion, for example, an uncomplete inclusion of K.

**Figure 2:**
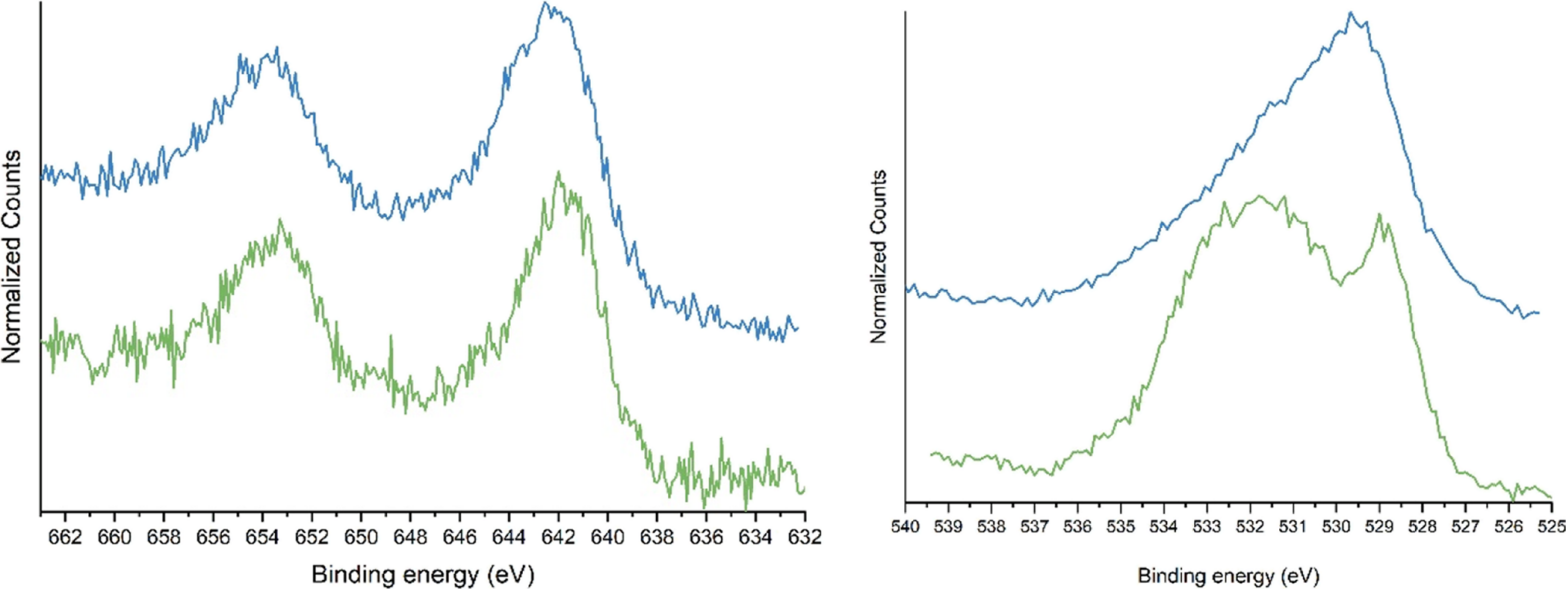
Mn 2p (left) and O 1s (right) XP spectra for the sample LKMC FSP (in green) and COP (in blue).

XP spectra (Figure 2) underline a significant effect of the preparation method on the surface composition. At first, besides lattice oxygen (components around 529–530 eV), a significant contribution due to other surface oxygen species can be observed. This is particularly evident for the FSP sample, in which surface oxygen species contribute with a broad signal centered at 532–533 eV; the peak position and shape are attributed to surface active oxygen species. For the COP catalyst, the relative amount of active oxygen seems less relevant and a tail at higher binding energies originates. The different shape can also be attributed to the presence of non-perovskitic oxides (contributing around 530 eV) because different oxygen species are responsible for different contributions in the O 1s XP spectrum range.

La3d peaks positions (834.2 and 838.2 eV) and shape (shake-up contributions at higher binding energies) indicate that lanthanum ions are present in the 3+ oxidation state;^19^ whereas a slight broadening for La3d_5/2_ in the COP sample suggests the presence, beside perovskite, of La (833.9–834.4 eV) of hydroxylic species, (La(OH)_3_ and LaOOH at about 835 eV).

The Mn 2p region was recorded to identify the Mn oxidative state, and the experimental evidences support the presence of mainly Mn(III) as the 2p_3/2_ is centered at 641.7–642.1 eV.^20^ The spin–orbit splitting is compatible with the species considered, with a Δ = 11.3 eV.^21^ Presence of the Mn(IV) ion cannot be well established due to the proximity of its peak to that of the Mn(III) ion; however, H_2_-TPR (temperature-programmed reduction) tests suggest its presence, shown later.^22^

The Co 2p peak shape and position are consistent with the presence of Co(III). No trace of K is visible from XP spectra in the samples probably due to the low potassium amount.

The fitting procedure was performed through a Shirley-type background subtraction and considering Gaussian fitting curves.^23^ The amount of surface oxygen is higher than the nominal value for both samples; this is consistent with the presence of hydroxyl groups and surface-active species suggested by the peak shape. Consistent with the peak shape, the surface over stoichiometry is more relevant in the FSP sample. The preparation procedure strongly affects the surface composition: La is segregated on the surface independent of the production procedure, but COP favors Mn surface segregation and FSP favors the presence of cobalt. Interestingly, the presence of K is only observed in the COP sample, and it is not detected by XP spectroscopy. A comparison of the compositions obtained with X-ray photoelectron spectroscopy (XPS) (surface analysis) and EDX (bulk analysis) techniques is reported in Table 1 and shows that the results are similar for the FSP sample, although differing from the nominal composition. The reason for such a behavior lies in the surface segregation of Co and the tendency of K to accumulate in the bulk rather that on the surface. Unlike the FSP sample, the COP one shows a different tendency in the metal cation distribution due to the scarce inclusion of K in the structure.

**Table 1:** XPS and EDX Atomic Compositions (%) Obtained for LKMC FSP and COP^a^

sample	type	La	K	Mn	Co	O	K/La	Co/Mn	(Mn + Co)/(La + K)	O/(La + K + Mn + Co)	Ratio lattice oxygen: surface oxygen (by XPS integration)
LKMC FSP	XPS	11.8	0.0	9.6	2.2	76.5	0.0	0.2	1.0	3.3	0.2
		50.2	0.0	40.6	9.2						
	EDX	12.1	0.4	10.2	2.3	75.1	0.0	0.2	1.0	3.0	
		48.7	1.6	41.0	9.3						
LKMC COP	XPS	19.1	0.0	18.2	0.0	62.6	0.0	0.0	1.0	1.7	0.5
		51.2	0.0	48.8	0.0						
	EDX	12.5	0.0	10.8	1.2	75.4	0.0	0.1	1.0	3.1	
		50.9	0.0	44.1	5.0						
nominal		18.0	2.05.0	18.0	2.05.0	60.0	0.1	0.1	1.0	1.5	
		45.0		45.0							

a In the cation columns, the first value is the one obtained considering oxygen also and the second one is obtained as cation-only compositions. These are reported in order to emphasize the cation surface segregation phenomena.

**Figure 3:**
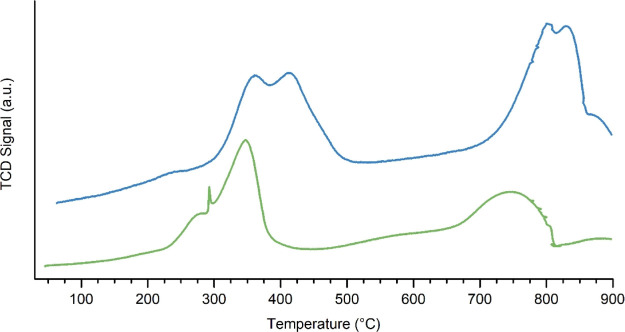
H_2_-TPR profile of the samples: in green, LKMC FSP and in blue, LKMC COP.

H_2_-TPR analysis was carried out to assess information about the reducibility of surface and bulk cations. As depicted in Figure 3 and reported in the quantitative Table 2, H_2_-TPR profiles differ from one sample to the other, giving the general impression that LKMC COP is less reducible than its analogue prepared by FSP. H_2_-TPR curves show a group of peaks around 300–450 °C and another one around 700–850 °C (Figure 3). The signals at lower temperature, according to literature sources, are assigned to the reduction of Mn(IV) to Mn(III)^24^ and those at higher temperature are related to the extensive reduction of Mn(III) to Mn(II).^24^ Cobalt reduction was observed to occur in two steps: from Co(III) to Co(II) at around 440 °C and from Co(II) to Co(0) at about 600 °C;^25−30^ reduction temperature, however, is deeply affected by the perovskite composition and the preparation procedure.^31,32^

**Table 2:** H_2_-TPR Results: Experimental Hydrogen Uptake Compared with the Expected and Fitting (Italics) Results^a^

	*T*_max_ (°C)	H_2_ mol consumed (·10^–3^)		species being reduced	stoichiometric coefficient	electrons involved	*n* of cation/mole perovskite (·10^–3^)	*n* of H_2_/g expected (·10^–3^)
LKMC FSP				Mn(III)	0.8 + 0.9(Mn^4+^)	1	3.87	1.94
*peak 1*	*309.7*	*0.97*		Mn(IV)	0.1	1	0.431	0.22
	*348.3*	*0.64*						
*peak 2*	*646.5*	*0.44*		Co(III)	0.1	3	0.431	0.65
	*745.8*	*0.60*						0.22
								0.43
*SUM*		2.65		SUM				2.80
LKMC COP								
*peak 1*	*351.4*	*0.36*						
	*414.4*	0.70						
*peak 2*	*803.3*	*0.08*						
	*837.6*	*0.84*						
*SUM*		1.98						

a The calculations for the expected amounts are reported on the right-hand side of the table.

The expected hydrogen consumption is determined considering the reduction of Mn(IV) to Mn(II) and of Co(III) to Co(0), and the relative quantities of such cations are obtained in stoichiometry (Mn^4+^ is present due to K substituting La), and it is equal to 2.80 mol/g (see right-hand side of Table 2). This value is compared to experimental hydrogen consumption, reported as number of moles consumed. From a first comparison between hydrogen consumed and expected (this being the last value constant for both samples), the FSP sample shows an almost perfect agreement (the difference is approx. 5%), whereas the COP sample consumes only 75% of the hydrogen theoretically needed to reduce it completely (1.98 mmol vs 2.80). This incomplete reduction together with the shift of the reductive reactions to higher temperatures suggests that COP is less reducible than FSP. The shape of the peaks and their position change with the preparation method, and their broadness indicates the presence of multiple species not equivalent in terms of reducibility.^33^ The different thermal stories of the samples have led to a different morphology of the samples and therefore to a different behavior when reduction is performed. Indeed, the FSP approach usually does not imply a following thermal treatment, which is instead very common in COP preparation. COP decreased reducibility might thus not only be the result of a different composition (different amount of K in the perovskite cell and different surface composition) but also of sintering phenomena occurred during calcination. The following table includes both the information about the peak (temperature, hydrogen consumption, and comparison between experimental and theoretical values) and the results of the fitting procedure performed on the peaks obtained (as reported in Supporting Information). The relative areas reported below show that the first peak, compatible with Mn(IV), Mn(III), and Co(III) reduction, is actually attributed to two distinct components, not directly assigned to any of such species because of the non-correspondence between stoichiometric coefficients and the relative areas. For this reason, we can conclude that the same species is actually present in different chemical environments and so suffers from different thermal behaviors in a reductive atmosphere. However, because the area of the first peak is quite large and not compatible with the small actual content of Mn(IV), Mn(III) is thought to reduce at a lower temperature than expected because Mn(IV) activates Mn(III) reduction. Moreover, because the first peak is attributed to easily reducible species, the larger area is observed for the FSP sample and therefore richer in Mn(IV). Also, some active oxygen species can contribute a small amount to the first reduction processes observed at low temperature (approximately 200 °C and below), as reported in the literature.^34^ Such species are more abundant in the FSP sample, as confirmed by XP spectra of the O 1s region.

**Figure 4:**
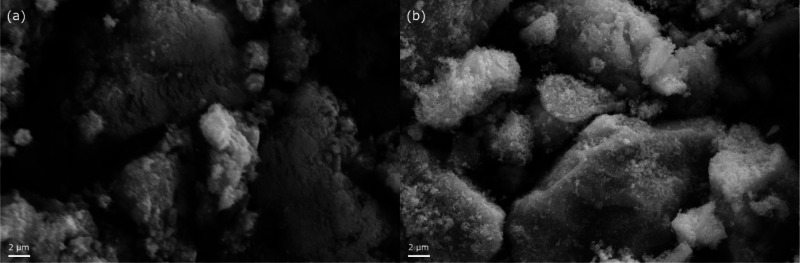
SEM images of LKMC FSP (a) and LKMC COP (b).

Representative SEM images are reported in Figure 4. The pictures show the formation of macro-aggregates with a diameter of several microns, covered with small particles which are homogeneous in size (few nanometers) and distribution in both samples. A significant difference is the tendency of the LKMC FSP sample to form aggregates of considerable size, whereas the LKMC COP sample tends to form both big and small aggregates. In particular, small aggregates appear as surface decoration on more compact, bigger particles. This can be due to the different thermal treatments which the samples have undergone in the preparation (i.e., higher temperatures for the LKMC FSP sample).

#### 3.3.2 Catalytic Activity

**Figure 5:**
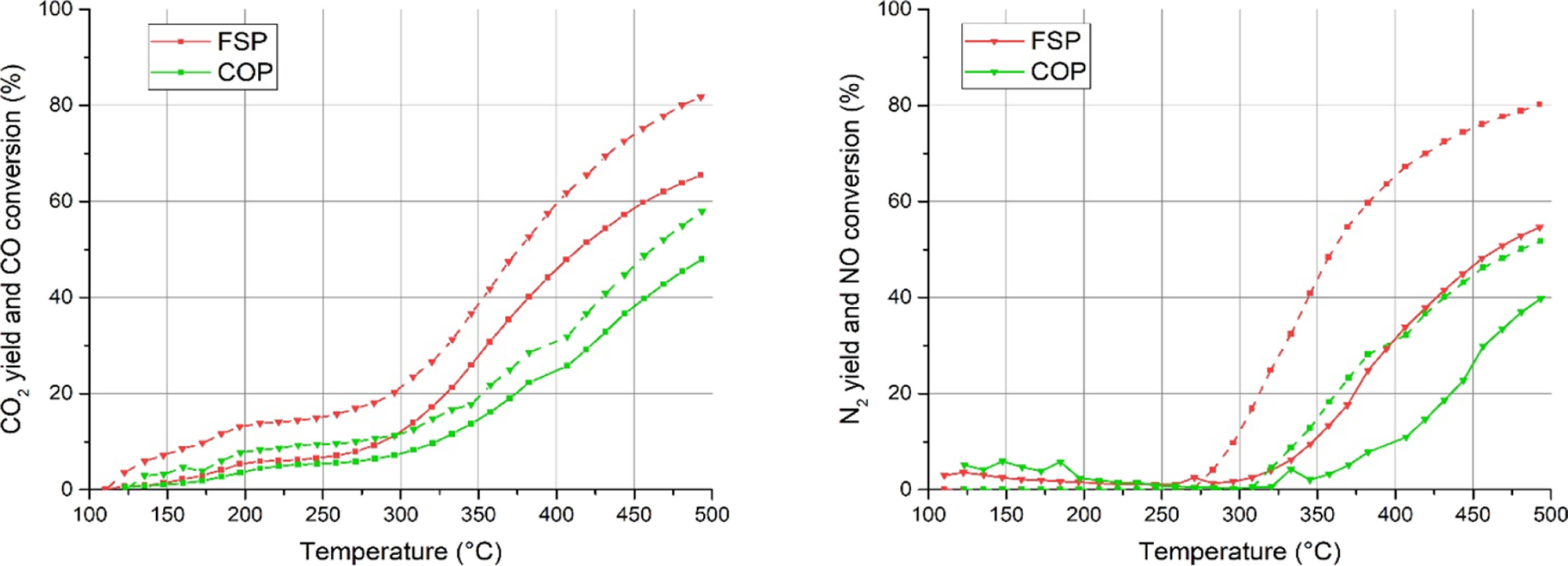
CO–NO mixture reactivity. CO_2_ and N_2_ yields are represented with solid lines and CO and NO conversion is represented with dashed lines.

The catalytic test suggests that the FSP sample gives better results in CO-assisted NO reduction (Figure 5) due to the inclusion of K in the structure, which leads to Co, instead of Mn, surface segregation. Catalytic activity starts being noticeable above 300 °C if we consider NO conversion. However, when CO oxidation is observed, a plateau of activity is seen in the same low temperature region (below 300 °C), with no significant NO reduction. This behavior suggests the involvement of surface oxygen species in CO oxidation, with no intervention from NO as an oxidant (suprafacial oxidation mechanism). As long as NO is not used as an oxidant (no appreciable conversion is detected), such mechanism is believed to take place when CO is actually converted to CO_2_. When NO conversion is also detected, the suprafacial oxidation mechanism decreases in favor of the mechanism commonly known as CO-assisted NO reduction.

**Figure 6:**
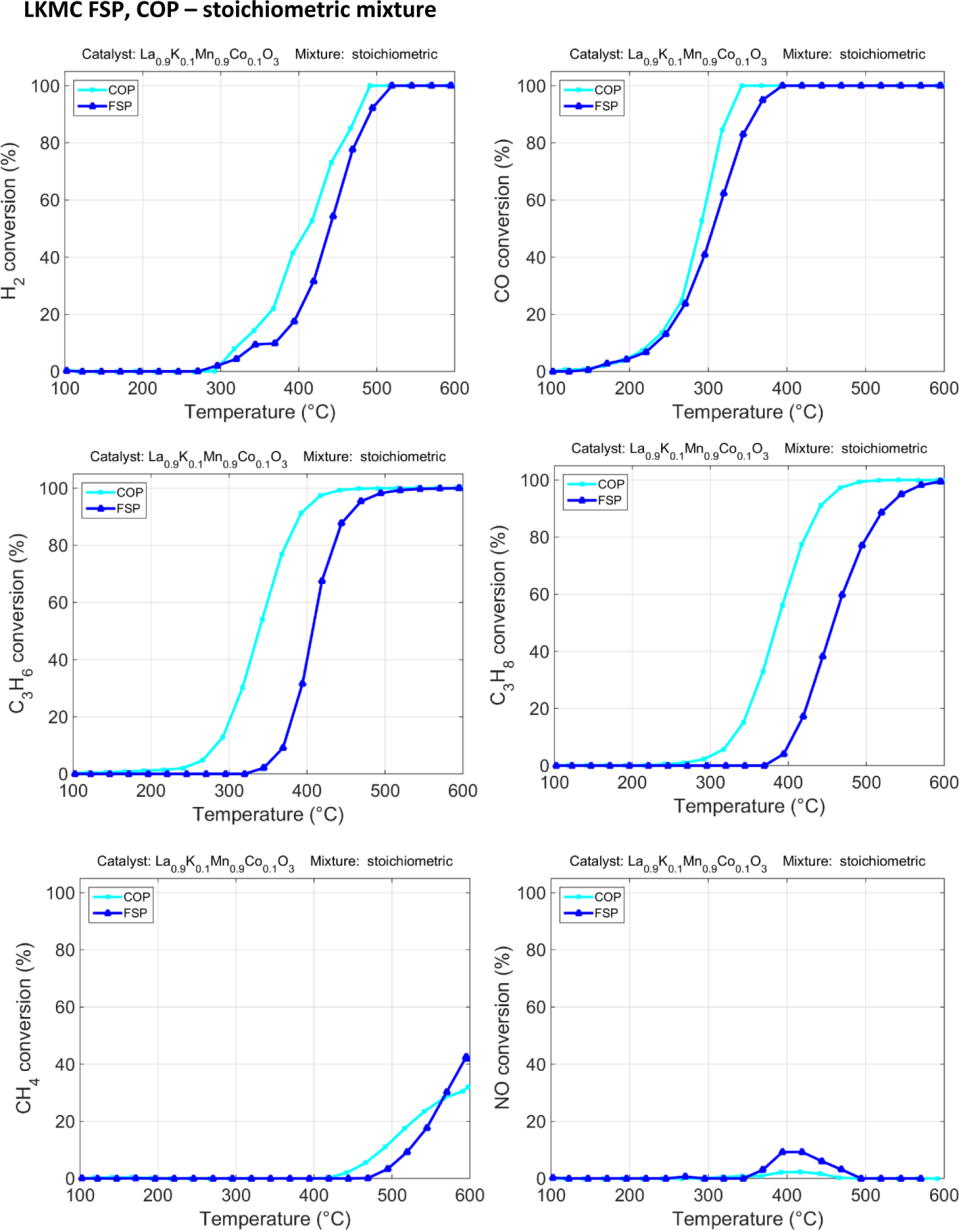
Stoichiometric mixture catalytic tests for LKMC FSP and COP.

**Figure 7:**
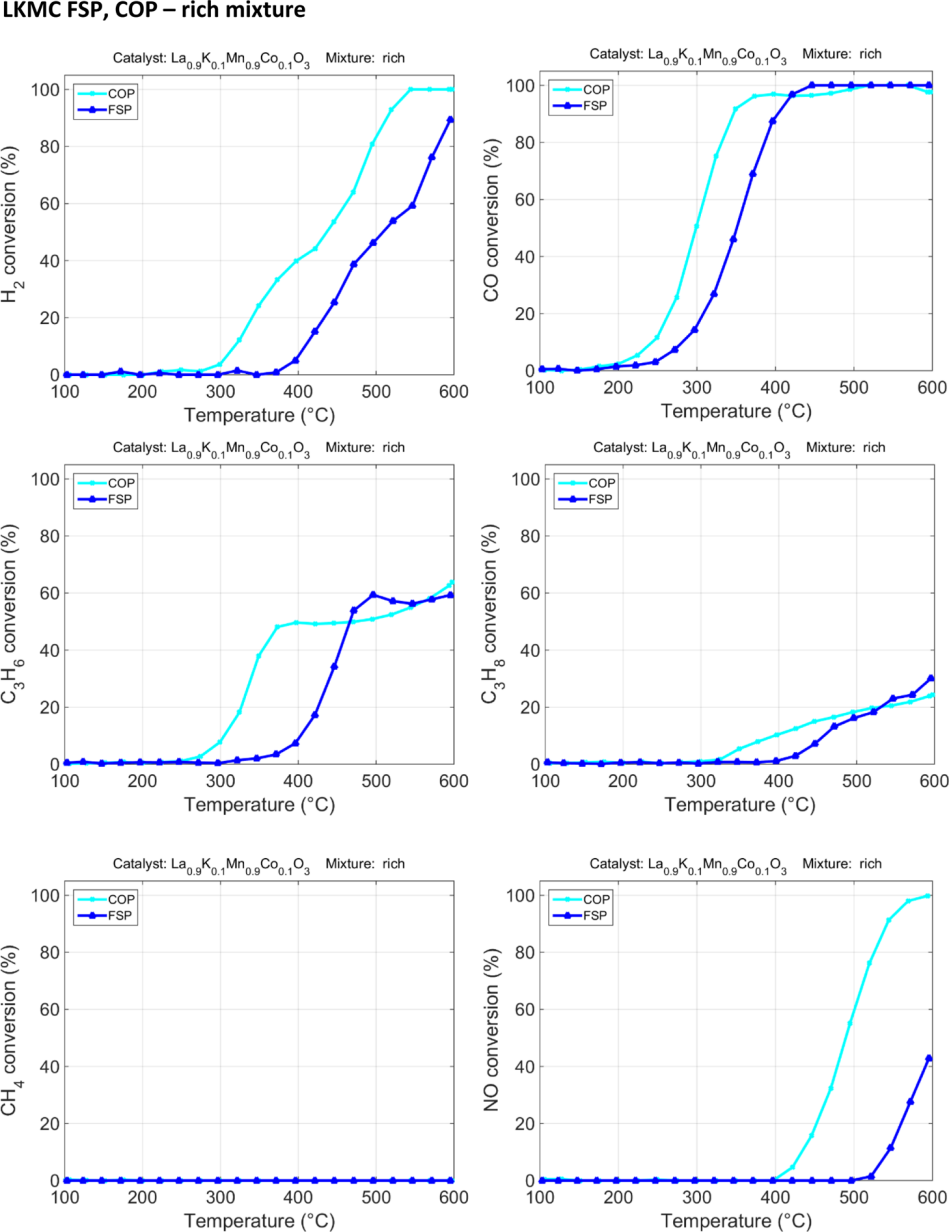
Rich mixture catalytic tests for LKMC FSP and COP.

Also, in the stoichiometric complex mixture, the two catalysts show different activities but the LKMC sample synthesized by COP has a high activity compared to the one synthesized by FSP (see Figures 6 and 7, O_2_ consumption is reported in the Supporting Information S8).^35^

The COP sample surface, in contrast to the FSP sample one, is richer in Mn(IV) (according to XPS—Table 1, surface composition), a well-working oxidant, which, indeed, allows to achieve good performances in a complex mixture. Propene is being converted at 270 °C for the sample LKMC COP, whereas the other sample converts it above 350 °C. A positive effect of temperature on the reaction rate is evident for propane oxidation between 300 and 450 °C, resulting in an enhancement of approximately 30% in conversion for LKMC FSP. Interestingly, under stoichiometric conditions, LKMC synthetized via FSP and COP shows a comparable temperature effect on the methane oxidation reaction rate.

As regards NO, the presence of O_2_ in stoichiometric amount prevents any significant NO reduction: it is occurring between 350 and 500 °C, reaching a maximum conversion of 9% for the FSP sample and 2.5% for the COP one. This is consistent with the mechanism proposed for perovskite that considers the interaction between NO and the oxygen vacancies present on the perovskite surface as the rate determining step for NO reduction.^5^

The profile of O_2_ consumption indicates the minimum temperature at which the catalyst is active that is approximately 200 °C for both samples under stoichiometric conditions. Above 400 °C, as regards the COP sample, oxidation of the HCs, H_2_, and CO is overall higher than that via FSP and the available O_2_ is totally consumed, not supplemented by NO reduction, activated in negligible way.

Concerning rich mixture testing conditions, in the range between 200 and 300 °C, the activated reactions are the oxidation of CO, H_2_, and C_3_H_6_. The LKMC COP sample looks much more active than its FSP obtained counterpart. In particular, a lower ignition temperature for C3 is observed for the COP sample. This sample is able to ignite for C3 reagents at about 250 °C, whereas the FSP sample exhibits ignition temperatures of 350 °C for C_3_H_6_ and 394 °C for C_3_H_8_. In COP activity profiles, propylene is totally converted at 491 °C, whereas FSP reaches maximum conversion at 545 °C. At 417 °C, propane conversion is 78% for the COP sample and only 17% for FSP. The conversion of propene is well below the values achieved in the stoichiometric case, explained by the total consumption of O_2_ that limits the oxidation of the hydrocarbons surviving that temperature.

Above 400 °C in the COP catalyst, all the available oxygen is exhausted; conversely, the LKMC FSP sample achieves the complete conversion of O_2_ at 500 °C.

When the total consumption of oxygen is achieved, NO is reduced, and it reaches complete conversion at 600 °C for LKMC COP, while for LKMC FSP, the conversion remains rather limited compared to the other catalysts (conversion of approx. 43%).

As regards rich conditions, evaluating O_2_ consumption is a useful way to sum up the activity of each sample and define which reactions are taking place. For the LKMC COP sample, at temperatures below 250 °C, the prevalent reaction is CO oxidation. On raising the reaction temperature, other oxidations are involved, such as H_2_, C_3_H_6_, and C_3_H_8_. Over 400 °C, all the oxygen available is depleted and the catalyst is exposed to a gaseous mixture containing oxidation products and unconverted CO, H_2_, and hydrocarbons, together with a high amount of steam (10%) and CO_2_ (15%). Therefore, at this temperature (400 °C), NO reduction begins and will be completely accomplished at 600 °C. For the LKMC FSP sample, oxygen conversion starts at 200 °C with CO oxidation. At temperatures higher than 300 °C, H_2_ and C_3_H_6_ are also reduced.

Summing up the behavior of the samples, because the mixture has run out of oxygen over 400 °C, the oxidation occurs exploiting oxygen derived from NO, which acts as an oxidant while getting reduced. This rationalization also explains the two-step oxidation of hydrogen probably due to this shift in the oxidant used. Methane does not get oxidized under rich conditions with any samples, which are therefore inactive to such a reagent in a slightly reducing environment (defect of oxygen according to reaction stoichiometry).

The specific surface area has been measured for each sample, but it does not actually affect the performance as expected: the COP sample suffers from aggregation and sintering due to thermal treatment, resulting in low surface areas, whereas the FSP sample area is roughly twice as much. This means that catalytic performance trend mainly depends on chemical and structural features of both the material surface and bulk.

## 4 Conclusions

In this article, we compare the chemical, structural, and morphological properties of La_0.9_K_0.1_Mn_0.9_Co_0.1_O_3_ catalyst powders, which were industrially obtained with two different processes: FSP and COP. Roughly speaking the catalysts seem very similar in the composition and structure. A detailed comparison, however, reveals that the degree of K-insertion inside the perovskite cell is process-dependent and the effects are expected on cell deformation, ion mobility, and surface composition. La and Mn segregate on the surface in the COP catalysts, while Co is surface-accumulated in the FSP catalyst. The different surface composition affects the reactivity; Brunauer–Emmett–Teller specific surface area differences, moreover, cannot be claimed as the reason for the different activity.

In the CO-assisted NO reduction, LKMC FSP is performing better than LKMC COP whereas in the complex mixture, the opposite is observed. The higher activity of FSP is related to the presence of oxygen active species on the perovskite surface and to a suprafacial oxidation mechanism. In the complex mixture, in contrast, the surface composition and the Mn-segregation seem to play a major role. Consistently, the results of the catalytic testing reveal that activity is quite different between the two catalysts, with a lower ignition temperature for CO, N_2_, and propene oxidation with the COP-obtained sample.

As regards NO reduction, the presence of O_2_ in stoichiometric amount prevents any significant NO reduction. The profile of O_2_ consumption indicates the minimum temperature at which the catalyst is active that is approximately 200 °C for both samples under stoichiometric conditions.

Concerning rich mixture testing conditions, the most relevant result is the NO reduction that is measured in the presence of depleted O_2_, considered as the actual obstacle for NO: when the total consumption of oxygen is achieved, NO is reduced, and it reaches complete conversion at 600 °C for LKMC COP.

For these reasons, the compositions of the bulk and surface are quite significative toward catalytic performances, in particular concerning the concentration of active oxygen species able to undergo the Mars-van Krevelen vacancy mechanism and the B cation distribution along the material depth. Interestingly doping at the A-site is able to deeply alter the mobility of the metal cation throughout the structure, resulting in different, and in some cases, worse catalytic activities.

A final remark on economic considerations must be given. In general, production cost estimates in FSP manufacture are difficult due to the variability of the raw material prices and geographically varying labor costs; the uncertainties in raw material price estimates being the main influent factor on the total production costs, which can be estimated below 100 EUR/kg, according to previous studies in the literature.^36^ Possible optimization margins therefore imply raw material-supply costs low-cost precursor solutions with higher metal-to-carbon rations, and a better utilization of the heat generated during combustion and strategies for more efficient flame quenching.^36^ Considering that one of the main costs of commercial catalysts is noble metals, perovskites provide a cheap alternative in terms of material cost and production.

The catalysts in this paper have been tested in their fresh state, but commercially viable catalysts must survive harsh hydrothermal ageing conditions at high temperature (>950 °C). Therefore, we consider that the next step is to optimize these materials to survive these ageing conditions, utilising the characterisation methods described in this paper to better understand the ageing process.

As a general conclusion comprising both Part 1 and Part 2, COP approach seems less effective in delivering an efficient dopant inclusion probably due to the difficulty in regulating finely the pH in a complex system of many different metallic cations in solution. This leads to important consequences in the composition distribution and therefore in the catalytic activity and selectivity. However, the extent of structural modifications due to the preparation approach greatly varies between different compositions..

## Supporting Information Available

Comparison based on the turn-over rate, H_2_-TPR profile fitting for LKMC FSP and COP, the description of the catalytic setup, and inductively coupled plasma composition results (PDF)
