# Supplementary material for: Industrially Produced Fe- and Mn-Based Perovskites: Effect of Synthesis on Reactivity in Three-Way Catalysis: Part 2
Source: ACS Omega. 2021 Sep 14;6(38):24316–24. doi: 10.1021/acsomega.1c02132 (PMC8482402; doi:10.1021/acsomega.1c02132)
Supplement: Supplementary file 1 — ao1c02132_si_001.pdf [file ao1c02132_si_001.pdf]

Industrially Produced Fe- and Mn-based perovskites: Effect of Synthesis on Reactivity in Three-Way Catalysis. Part 2

Elena Brusamarello[a], Cataldo Blonda[b], Cristina Salazar-Castro[c], Paolo Canu[b], Antonella Glisenti [a,d]*

[a] Dept. of Chemical Sciences, University of Padova, - Via F. Marzolo, 1, 35131, Padova, Italy.

[b] Dept. of Industrial Engineering, University of Padova, - Via F. Marzolo, 9, 35131, Padova, Italy.

[c] L'Urederra Foundation, Perguita Industrial Area, No. 1 street, CP: 31210 Los Arcos (Navarra) Spain

[d] CNR-ICMATE, INSTM - Via F. Marzolo, 1, 35131, Padova, Italy.

Supplementary material

Comparison based on turn-over rate

With the purpose of accounting for the dramatic differences in specific surface among the same materials produce by different synthetic routes, we tried to reformulate the comparison in terms of turn-over rate (TOR), defined as the number of reagent molecules consumed per single site, in the unit time. Unfortunately, that require knowledge of the surface site availability for several species in the complex mixture, without a precise knowledge of the reaction mechanism. In addition, TOR is expected to vary along the catalyst bed. Given these uncertainties, we recast Figure 7 and 8 in terms of TOR for each species, averaged over the entire catalyst bed, calculated as:

$$TOR = \frac{-\Delta \dot{N}_i}{(\Gamma/\mathrm{NR})\ SSA\ m_{cat}}$$

Where $\dot{N}_i$is the molar flow rate (moles/s) of each reactant *i=1..NR=*H_2, O_2, *CO*, CH_4, C_3H_6, C_3H_8, *NO,* $\Delta\dot{N}_i$is the difference between outlet and inlet, Γ/NR the surface site density (moles/m^2) and SSA the specific surface areas (m^2/g_{cat}). The sites are assumed to be shared among the NR reactants (including O_2); their surface density estimated as 1.5 10^{-5} moles/m^2. While the above assumptions on the available sites (surface density and occupancy) can be arbitrary, the estimated TOR introduces the active surface of the materials in their comparison.

The ranking among the 3 materials is much sharper, because of the differences in the SSA. For all the reactants, the ranking is unambiguously LCFC-COP>LFC-FSP>LCFC-FSP for both stoichiometric and rich mixtures (see Part 1), simply because of the reverse order in the specific surface areas, i.e. LCFC-FSP>LFC-FSP > LCFC-COP. TOR plots also allow to reveal that the activity of each site follows the order CO≈O2 > H_2 >> HCs in stoichiometric mixtures, and CO > O_2 > H_2 > NO > C_3H_6 in the rich one.

Notwithstanding the approximation, the order of TOR calculated, between 10^{-1} and 10^{1} molecules/ (site s) is perfectly in line with the indications of Somorjai[1] for this interval of temperature.

Peak Analysis LKMC FSP

Data Set:[Book2]Sheet1!E"TCD Signal" Date:12/10/2020

BaseLine:BSpline

Chi^2=5,12298E+000 Adj. R-Square=9,87588E-001 # of Data Points=854

SS=4,31355E+003 Degree of Freedom=842

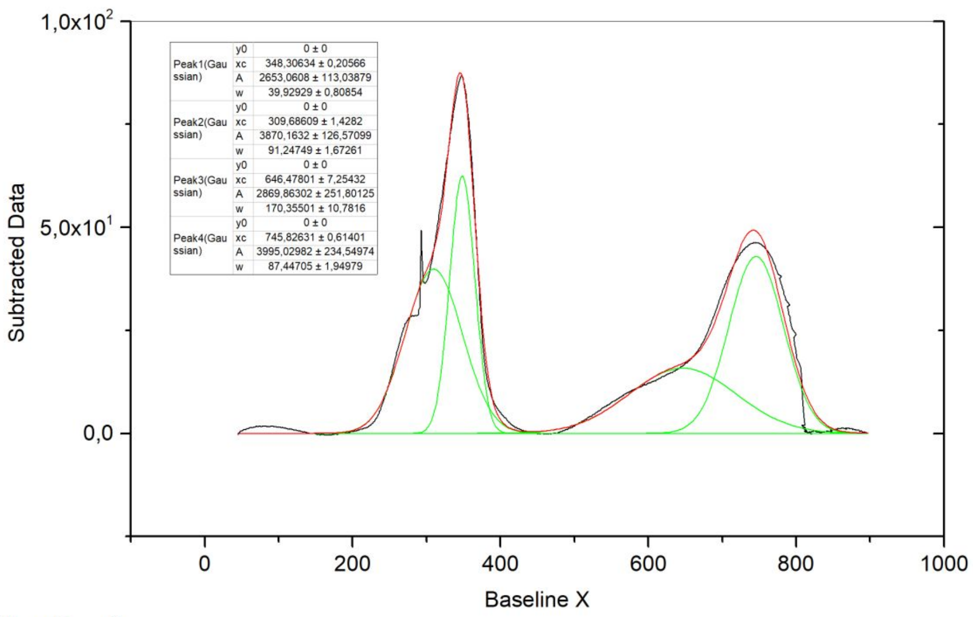

Fitting Results

Peak Index	Peak Type	Area Intg	FWHM	Max Height	Center Grvty	Area IntgP
1	Gaussian	2653,0608	39,92929	62,41995	348,30634	19,8178
2	Gaussian	3870,1632	91,24749	39,84521	309,68609	28,90929
3	Gaussian	2869,10179	170,35501	15,82611	646,47801	21,43158
4	Gaussian	3994,93738	87,44705	42,91831	745,82631	29,84133

Figure S1. H_2-TPR fitting results for LKMC FSP.

Peak Analysis

Data Set:[Book2]Sheet1!G"TCD Signal" Date:12/10/2020

BaseLine:Spline

Chi^2=2,84948E+000 Adj. R-Square=9,96470E-001 # of Data Points=827

SS=2,32803E+003 Degree of Freedom=817

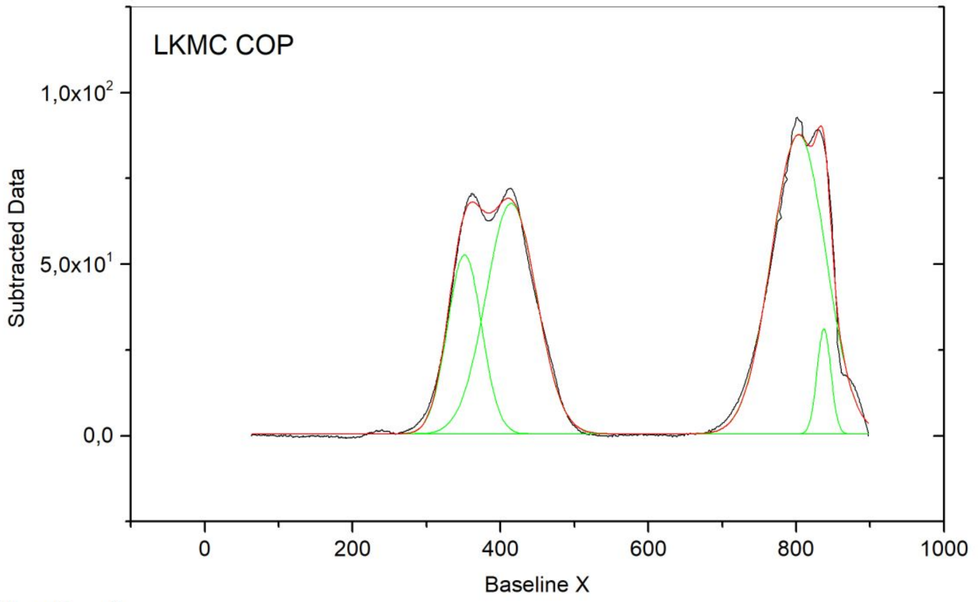

Fitting Results

Peak Index	Peak Type	Area Intg	FWHM	Max Height	Center Grvty	Area IntgP
1	Gaussian	3017,26302	54,33809	52,16469	351,38734	17,30875
2	Gaussian	5746,3911	80,37323	67,16632	414,41906	32,9646
3	Gaussian	7954,92694	86,07011	87,25239	803,36237	45,63403
4	Gaussian	713,42539	21,90454	30,59724	837,60877	4,09262

Figure S2. H_2-TPR fitting results for LKMC COP.

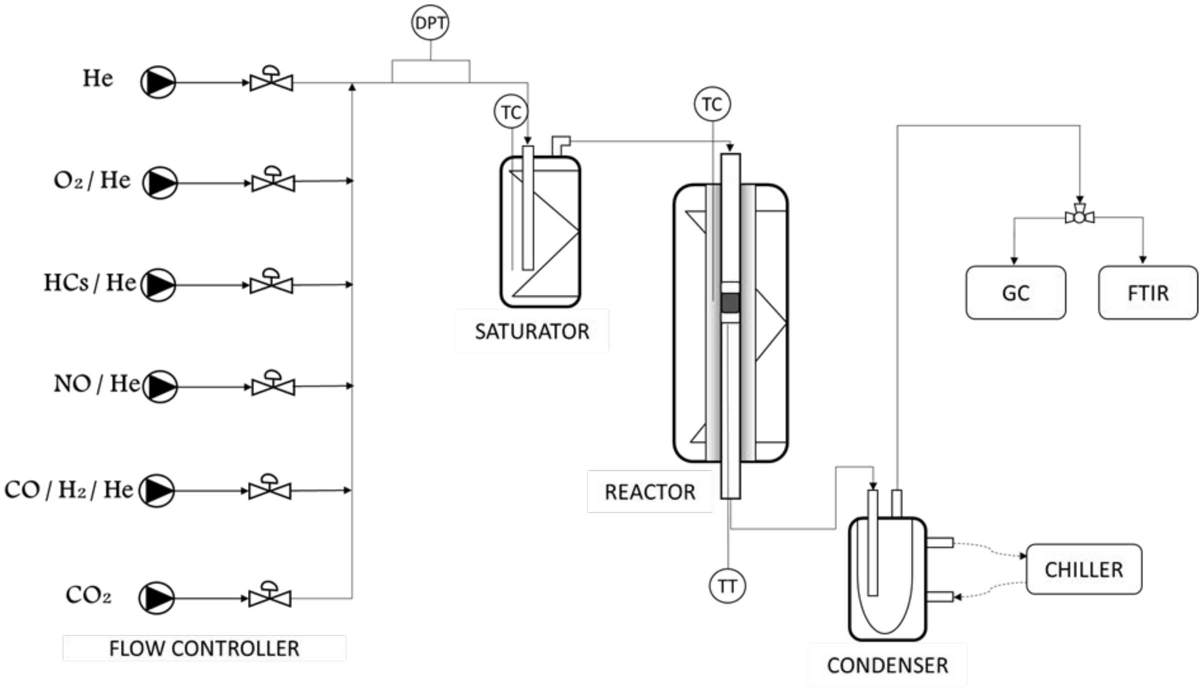

Figure S3. Catalytic setup for complex mixture testing.

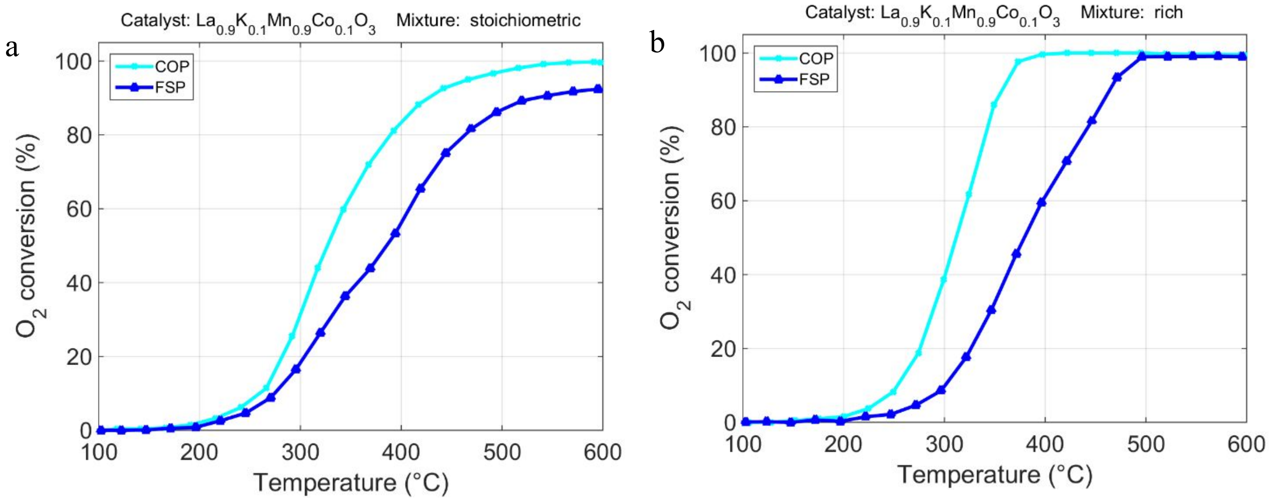

Figure S4. Stoichiometric (a) and rich (b) mixture catalytic tests for LKMC FSP, COP.

ICP analytic results

LKMC experimental ICP composition is in good agreement with the nominal one in both cases. Among dopants, Co is also more efficiently included in the structure as compared to K (Table S1).

		ICP results (%)	
	Nominal	**LKMC FSP**	**LKMC COP**
La	67.91	70.40	69.35
K	2.12	1.64	1.28
Mn	26.76	23.73	26.57
Co	3.21	4.23	2.79

Table S1. ICP composition of the samples compared to the nominal composition (only metal).

References

(1) Somorjai, G. A.; Li, Y. *Introduction to Surface Chemistry and Catalysis*, 2nd Editio.; Wiley, 2010.
